# Estimating Pack-Year Eligibility for Lung Cancer Screening Using 2 Yes or No Questions

**DOI:** 10.1001/jamanetworkopen.2023.27363

**Published:** 2023-08-07

**Authors:** Katharine A. Rendle, Jennifer P. Steltz, Sarah Cohen, Marilyn M. Schapira, Richard C. Wender, Justin E. Bekelman, Anil Vachani

**Affiliations:** 1 Department of Family Medicine & Community Health, Perelman School of Medicine, University of Pennsylvania, Philadelphia; 2 Penn Center for Cancer Care Innovation, Abramson Cancer Center, Philadelphia, Pennsylvania; 3 Division of Pulmonary and Critical Care, University of Pennsylvania School of Medicine, Philadelphia; 4 Department of General Internal Medicine, Perelman School of Medicine, University of Pennsylvania, Philadelphia; 5 Department of Radiation Oncology, Perelman School of Medicine, University of Pennsylvania, Philadelphia; 6 Corporal Michael J. Crescenz Veterans Affairs Medical Center, Philadelphia, Pennsylvania

## Abstract

This cross-sectional study describes the development and testing the accuracy of using 2 yes or no questions to estimate pack-year eligibility for lung cancer screening.

## Introduction

Lung cancer screening (LCS) for adults at high risk reduces lung cancer mortality by 16% to 20%.^1^ However, uptake of LCS remains low and unequal in the US.^2,3^ LCS differs from other types of cancer screening because eligibility assessment requires evaluation of lifetime smoking intensity (ie, pack-years). Although tobacco use (ie, current, former, or never) is well captured, calculated pack-years are often missing or inaccurate in electronic medical records (EMRs).^4^ Prior approaches to assessing pack-years have used open-ended questions, a format associated with increased respondent burden and lower response rates.^5^ There is a need for tools that simplify pack-year assessment while maintaining accuracy.^4^ In this cross-sectional study, we developed and tested the accuracy of using 2 yes or no questions to estimate pack-year eligibility for LCS.

## Methods

This study, approved by the University of Pennsylvania institutional review board (IRB), was embedded within a parent pilot trial aimed at increasing LCS at a multisite academic health care system (NCT04612946). The parent trial used EMR data to identify primary care patients aged 55 to 80 years with no history of LCS or lung cancer and listed as currently smoking from 2018 to 2020. Prior to trial enrollment, we invited identified patients to complete an LCS eligibility survey electronically or via telephone using a waiver of written consent approved by the University of Pennsylvania IRB because the research placed no more than minimal risk to the participants. The survey asked open-ended questions regarding total number of years and mean number of packs per day smoked to calculate pack-years. The survey also included the following 2 yes or no questions: (1) If you add up all the years when you regularly smoked cigarettes, have you smoked for 20 years or more of your life? (2) At any time in your life, did you regularly smoke 1 or more packs of cigarettes per day? We evaluated the sensitivity, specificity, positive predictive value (PPV), and negative predictive value (NPV) of the questions to estimate eligibility (ie, 20 or more pack-years) using 2 classifications of a positive response: (1) liberal (yes to at least 1 question) and (2) restrictive (yes to both questions). We calculated 95% CIs using the Clopper-Pearson method, with a 2-sided *P* < .05 considered statistically significant. Using the STROBE reporting guideline, we report results overall and stratified by sex, age, and race and ethnicity as documented in the patient’s EMR (due to known racial and ethnic disparities in lung cancer). All analyses were performed using Stata, version 17 (StataCorp LP).

## Results

Of 303 patients who completed the survey, 156 (51.5%) were aged 55 to 64 years, 176 (58.1%) were female, and 62 (20.5%) were Black or African American; 210 (69.3%) reported 20 or more pack-years (Table 1). Using the liberal classification (Table 2), the yes or no questions had 100% NPV and 100% sensitivity for estimating pack-year eligibility for LCS but lower specificity overall (18.3%) and within subgroups (range, 13.0%-25.6%). Overall specificity (79.6%) and PPV (90.5% [range within subgroups, 88.3%-92.8%]) increased using the restrictive classification, but sensitivity decreased (85.7%), particularly among adults aged 55 to 64 years (81.4%) or Black adults (75.6%).

**Table 1.** Participant Characteristics

Characteristic^a^	No. (%) (N = 303)
Age group, y	
55-64	156 (51.5)
65-74	125 (41.3)
≥75	22 (7.3)
Sex	
Female	176 (58.1)
Male	127 (41.9)
Race	
Asian	1 (0.3)
Black or African American	62 (20.5)
Native American	1 (0.3)
Pacific Islander	0
White	220 (72.6)
Other^b^	13 (4.3)
Unknown	6 (2.0)
Ethnicity	
Hispanic	4 (1.3)
Non-Hispanic	295 (97.4)
Unknown	4 (1.3)
Tobacco use	
Currently smokes	303 (100.0)
Smoking pack-years	
30-39	26 (8.6)
40-49	25 (8.3)
≥50	27 (8.9)
Missing pack-years	225 (74.3)
Smoking pack-years (self-reported in survey)^c^	
<20	93 (30.7)
20-29	35 (11.6)
30-39	46 (15.2)
40-49	53 (17.5)
≥50	76 (25.1)

^a^ All patient data unless otherwise noted were taken from the patient’s medical record at the time of enrollment.

^b^ Racial category is listed as “other” in the electronic medical record, and therefore no other information on patient race is available.

^c^ Participants were asked to answer the following open-ended questions to calculate pack-years: (1) Thinking about when you first started smoking regularly, how many total years have you smoked cigarettes across your lifetime? (2) Across your lifetime (on average), how many cigarettes have you usually smoked each day? There are 20 cigarettes in a pack. Lifetime pack-years calculated by multiplying each participant’s responses to the 2 questions and dividing by 20 (the number of cigarettes in a pack).

**Table 2.** Accuracy of Yes or No Questions for Estimating Pack-Year Eligibility for LCS^a^

Characteristic	Overall (N = 303)	Age, y		Biological sex		Race	
		<65 (n = 156)	≥65 (n = 147)	Female (n = 176)	Male (n = 127)	Black (n = 62)	White (n = 220)
**Simplified yes or no questions**							
Yes, to both questions, No. (%)	199 (65.7)	94 (60.3)	105 (71.4)	116 (65.9)	83 (65.4)	35 (56.5)	152 (69.1)
Yes, to only 1 question, No. (%)	87 (28.7)	55 (35.3)	32 (21.8)	53 (30.1)	34 (26.8)	24 (38.7)	58 (26.4)
No, to both, No. (%)	17 (5.6)	7 (4.5)	10 (6.8)	7 (4.0)	10 (7.9)	3 (4.8)	10 (4.6)
**Calculated pack-years (open-ended questions)**							
LCS eligible (≥20 pack-years), No. (%)	210 (69.3)	102 (65.4)	108 (73.5)	123 (69.9)	87 (68.5)	41 (66.1)	161 (73.2)
LCS ineligible (<20 pack-years), No. (%)	93 (30.7)	54 (34.6)	39 (26.5)	43 (24.4)	40 (31.5)	21 (33.9)	59 (26.8)
**Accuracy using liberal classification: positive indicates yes to at least 1 question**							
Sensitivity (95% CI), %	100.0 (98.3-100.0)	100.0 (96.5-100.0)	100.0 (96.6-100.0)	100.0 (97.0-100.0)	100.0 (95.8-100.00)	100.0 (91.4-100.0)	100.0 (97.7-100.0)
Specificity (95% CI), %	18.3 (11.7-27.5)	13.0 (6.3-24.9)	25.6 (14.3-41.6)	13.2 (6.3-25.3)	25.0 (13.9-40.7)	14.3 (4.6-36.7)	16.9 (9.3-28.8)
PPV (95% CI), %	73.4 (68.0-78.2)	68.5 (60.5-75.5)	78.8 (71.1-84.9)	72.8 (65.5-79.0)	74.4 (65.6-81.5)	69.5 (56.4-80.0)	76.7 (70.4-81.9)
NPV (95% CI), %	100.0 (80.5-100.0)	100.0 (59.0-100.0)	100.0 (69.2-100.0)	100.0 (59.0-100.0)	100.0 (69.2-100.0)	100.0 (29.2-100.0)	100.0 (69.2-100.0)
**Accuracy using restrictive classification: positive indicates yes to both questions**							
Sensitivity (95% CI), %	85.7 (80.2-89.8)	81.4 (72.6-87.9)	89.8 (82.5-94.3)	84.6 (77.0-90.0)	87.4 (78.5-92.9)	75.6 (60.0-86.5)	87.6 (81.5-91.9)
Specificity (95% CI), %	79.6 (70.1-86.6)	79.6 (66.7-88.4)	79.5 (63.9-89.5)	77.4 (64.1%-86.7%)	82.5 (67.4-91.5)	81.0 (58.3-92.8)	81.4 (69.3-89.4)
PPV (95% CI), %	90.5 (85.5-93.8)	88.3 (80.0-93.4)	92.4 (85.4-96.2)	89.7 (82.7-94.1)	91.6 (83.2-96.0)	88.6 (72.8-95.7)	92.8 (87.4-96.0)
NPV (95% CI), %	71.2 (61.7-79.0)	69.4 (56.8-79.6)	73.8 (58.5-84.9)	68.3 (55.5-78.9)	75.0 (60.1-85.7)	63.0 (43.4-79.0)	70.6 (58.7-80.2)

Abbreviations: LCS, lung cancer screening; NPV, negative predictive value; PPV, positive predictive value.

^a^ Age, sex, and race are as reported in the electronic medical record. For race, we limited comparison with the most common racial groups in our sample (Black adults vs White adults).

## Discussion

Response to 2 yes or no questions showed high sensitivity for estimating pack-year eligibility for LCS, helping to identify pragmatic solutions for overcoming the challenge of assessing pack-years in routine practice.^6^ We observed differences in sensitivity and specificity between liberal and restrictive classifications of positive response. However, these differences might also increase the range of utility of these questions in practice. The restrictive classification could be used to equitably identify patients for direct referral to shared decision-making for LCS given the high PPV overall (90.5%) and within each subgroup (88.3%-92.8%), whereas the liberal classification could be used to efficiently exclude patients from LCS outreach given the consistently high NPV (100.0%). Our study is limited by its sample size, single site, and exclusion of years since quitting among adults who formerly smoked. Future work, including testing in a larger national sample of participants aged 50 to 80 years, could expand on these findings.

## Notes

Supplement.
Data Sharing Statement
