# Supplementary material for: Estimating Pack-Year Eligibility for Lung Cancer Screening Using 2 Yes or No Questions
Source: JAMA Netw Open. 2023 Aug 7;6(8):e2327363. doi: 10.1001/jamanetworkopen.2023.27363 (PMC10407683; doi:10.1001/jamanetworkopen.2023.27363)
Supplement: Supplement. — Data Sharing Statement [file jamanetwopen-e2327363-s001.pdf]

Data Sharing Statement

Rendle. Estimating Pack-Year Eligibility for Lung Cancer Screening Using 2 Yes or No Questions. *JAMA Netw Open*. Published August 07, 2023.
doi:10.1001/jamanetworkopen.2023.27363

Data
Data available: Yes
Data types: Deidentified participant data, Data dictionary
How to access data: katharine.rendle@pennmedicine.upenn.edu
When available: With publication

Supporting Documents
Document types: None

Additional Information
Who can access the data: Researchers whose proposed use of the data has been approved
Types of analyses: For a specific purpose
Mechanisms of data availability: With a signed data access agreement
Any additional restrictions: Contingent upon local IRB approval
